# Zeaxanthin Isolated from *Dunaliella salina* Microalgae Ameliorates Age Associated Cardiac Dysfunction in Rats through Stimulation of Retinoid Receptors

**DOI:** 10.3390/md17050290

**Published:** 2019-05-14

**Authors:** Farouk Kamel El-Baz, Rehab Ali Hussein, Dalia Osama Saleh, Gehad Abdel Raheem Abdel Jaleel

**Affiliations:** 1 Plant Biochemistry Department, National Research Centre (NRC), 33 El Buhouth St. (Former El Tahrir St.), Dokki, Giza P.O.12622, Egypt; fa_elbaz@hotmail.com; 2 Pharmacognosy Department, National Research Centre (NRC), 33 El Buhouth St. (Former El Tahrir St.), Dokki, Giza P.O.12622, Egypt; 3 Pharmacology Department, National Research Centre (NRC), 33 El Buhouth St. (Former El Tahrir St.), Dokki, Giza P.O.12622, Egypt; doabdelfattah@yahoo.com (D.O.S.); gehad_abougharam@yahoo.com (G.A.R.A.J.)

**Keywords:** zeaxanthin, carotenoids, *Dunaliella salina*, aging, cardiac dysfunction, retinoid receptors

## Abstract

Retinoids are essential during early cardiovascular morphogenesis. However, recent studies showed their important role in cardiac remodeling in rats with hypertension and following myocardial infarction. The present study aimed to investigate the effect of zeaxanthin heneicosylate (ZH); a carotenoid ester isolated from *Dunaliella salina* microalgae, on cardiac dysfunction ensuing d-galactose injection in rats. Rats injected with d-GAL (200 mg/kg; I.P) for 8 weeks were orally treated with ZH (250 μg/kg) for 28 consecutive days. Results showed that d-GAL injection caused dramatic electrocardiographic changes as well as marked elevation in serum levels of homocysteine, creatinine kinase isoenzyme and lactate dehydrogenase. A reduction in the cardiac contents of glucose transporter-4 and superoxide dismutase along with the elevation of inducible nitric oxide synthetase and interleukin-6 was also noticed. Oral administration of ZH significantly improved the above mentioned cardiac aging manifestations; this was further emphasized through histopathological examinations. The effect of ZH is mediated through the interaction with retinoid receptor alpha (RAR-α) as evidenced through a significant elevation of RAR-α expression in cardiac tissue following the lead of an in silico molecular docking study. In conclusion, zeaxanthin heneicosylate isolated from *D. salina* ameliorated age-associated cardiac dysfunction in rats through the activation of retinoid receptors.

## 1. Introduction

During embryogenesis, retinoids, the metabolic products of carotenoids, play an essential role in the morphogenesis of the cardiovascular system. This role is mediated through the activation of the retinoic acid receptor (RAR) involved in signal transduction pathways regulating embryonic development, tissue homeostasis, and cellular differentiation and proliferation. However, previous studies have shown that retinoids have a major role in the cardiac remodeling process in hypertensive rats with myocardial infarction [1].

Carotenoids; plant pigments are bioconverted to retinol then to retinoic acid (RA) first in the intestine, then the liver, and finally in target cells. RA is vital for modulating a wide range of biological processes such as cell differentiation and proliferation, vision, bone formation, metabolism, and immunological processes [2]. RA is a potent transcriptional regulator and a natural ligand for the retinoic acid receptor (RAR) and retinoid X receptors (RXRs), which are DNA-binding transcriptional regulators. These receptors form homodimers (RXR/RXR) and heterodimers (RXR/RAR), which directly activate gene transcription, by binding to specific RA response elements in target gene promoter regions [3]. Therefore the investigation and development of retinoid derivatives as drugs that target RARs and RXRs are very promising for the treatment of a multitude of ailments [4].

*Dunaliella salina* Teodoresco; unicellular marine phytoplankton that belong to the phylum Chlorophyta [5] are considered to be the richest natural producer of massive carotenoids. However, β-carotene, pro-vitamin A, remains the major natural product harvested from *D. salina* (up to 1% dry weight). *D. salina* exhibited the growth inhibition activity and proapoptotic effects on human colon cancer cell lines, many types of cancer, and degenerative diseases in vitro and in vivo [6] probably due to their antioxidant and anti-inflammatory activities. The previous study held in our lab revealed a promising curative and prophylactic efficacy of *D. salina* against cardiac dysfunction in senile rats [7], which was an urge towards investigating the phytochemical constituent underlying this effect.

Hence, the present study aimed to investigate the beneficial effect of zeaxanthin heneicosylate (ZH); a major carotenoid isolated from *D. salina* on cardiac dysfunction associated with d-galactose (d-GAL)-induced aging in rats. To achieve this aim, electrocardiographs were recorded as well as assessment of serum cardiac function *viz.*, homocysteine (HS), creatinine kinase isoenzyme (CK-MB), lactate dehydrogenase (LDH) and glucose trasporter 4 (GLUT-4); cardiac oxidative stress biomarkers *viz*., superoxide dismutase (SOD) and cardiac inflammatory mediators *viz*., inducible nitric oxide synthetase (iNOS) and interleukin-6 (IL-6) were all performed for both treated and untreated groups. Histopathological changes in the cardiac tissue were recorded. Moreover, retinoid acid receptor alpha (RAR-α) gene expression was estimated in cardiac tissues.

## 2. Results

### 2.1. Isolation and Identification of ZH

**Figure 1:**
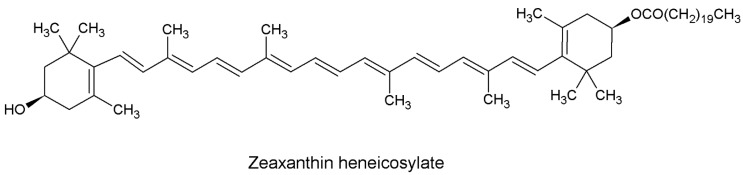
Chemical structure of zeaxanthin heneicosylate (ZH).

Repeated chromatographic analysis of *D. salina* led to the isolation of an amorphous orange compound. Spectroscopic analysis of the isolated compound revealed the following results: The UV/VIS in petroleum ether λmax: 423, 446, 475 (%III/II 25). ^1^H NMR (400 MHz, chloroform-*d*) δ ppm 1.18 (s, 3 H, H16,16’) 1.19 (s, 3 H, H17,17’) 1.72 (t, *J* = 2.53 Hz, 6 H, H18,18’) 1.97 (d, *J* = 3.20 Hz, 6 H, H20,20’) 1.98 (d, *J* = 3.85 Hz, 6 H, H19,19’) 2.06 (d, *J* = 8.00 Hz, 4 H, H2,2’) 2.34 (m, 4 H, H4,4’) 3.96 (t, *J* = 12.00, 8.00 Hz, 1 H, H3’) 4.02 (t, *J* = 12.00, 8.00 Hz, 1 H, H3) 6.08 (m, 2 H, H7,7’) 6.13 (m, 2 H, H8,8’) 6.17 (m, 2 H, H10,10’) 6.38 (sc, *J* = 10.82 Hz, 2 H, H15,15’) 6.43 (m, 2 H, H12,12’) 6.54 (m, 2 H, H14,14’) 6.61 (m, 2 H, H11,11’). ^13^C NMR (101 MHz, chloroform-*d*) δ ppm: 13.01 (C20) 13.03 (C20’) 13.08 (C19) 13.22 (C19’) 21.55 (C18) 21.59 (C18’) 27.70 (C16) 27.96 (C16’) 28.20 (C17) 28.29 (C17’) 30.76 (C1) 30.90 (C1’) 30.93 (C4) 33.37 (C4’) 36.73 (C2) 37.72 (C2’) 59.10 (C3) 67.11 (C3’) 123.26 (C7) 123.29 (C7’) 126.09 (C13) 126.70 (C13’) 126.89 (C10) 127.02 (C10’) 127.23 (C9) 127.25 (C9’) 127.57 (C11) 127.78 (C11’) 128.65 (C8) 128.73 (C8’) 128.82 (C12) 128.89 (C12’) 128.96 (C6) 129.02 (C6’) 129.17 (C5) 129.24 (C5’) 129.74 (C14) 129.84 (C14’) 130.77 (C15) 130.91 (C15’) 9.93 (C21”) 172.89 (C1”) 30.51 (C2”) 21.63–27.69 (C3”–C19”). ESI showed molecular ions [M − heneicosyl residue]^+^ at *m*/*z* = 568.426 and [M + H]^+^ *m*/*z* = 913.683 (Figure 1).

### 2.2. Molecular Docking Study

**Figure 2:**
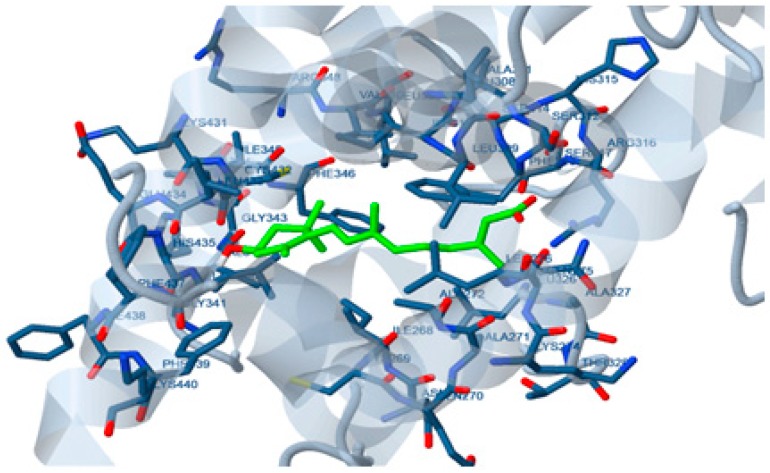
Docking of zeaxanthin metabolic product on retinoic acid receptor (RAR)-α.

In silico molecular docking showed that the estimated free energies of binding of 15-apo-zeaxanthenoic acid and 9’-apo-zeaxanthenoic acid; the metabolic analogues of retinoic acid on the interactive surface of RAR-α were −1.50 and +61.90 kcal/mol, respectively (Figure 2). Whereas, the estimated free energies of binding of 15-apo-zeaxanthenoic acid and 9’-apo-zeaxanthenoic acid on the interactive surface of RXR were −0.63 and +78.93 kcal/mol, respectively.

### 2.3. Pharmacological Study

#### 2.3.1. Acute Toxicity Study on Mice

Acute toxicity study with ZH was performed on mice. ZH has shown no acute toxicity indicating its high safety margin where all mice survived a single oral dose of up to 1g/kg.

#### 2.3.2. Effect of ZH on Age-Associated Cardiac Dysfunction in Rats

##### Effect of ZH on the Electrocardiographic (ECG) Measurements

Cardiac dysfunction was induced in rats by injection of d-GAL (200 mg/kg I.P) for 8 weeks. d-GAL injected rats were orally treated with ZH for four weeks after d-GAL injection. Rats were then anaesthetized with thiopental and ECG was recorded. d-GAL intraperitoneal injection showed dramatic changes in the ECG pattern of treated rats in the form of irregular rhythm of heartbeats, depressed ST height as well as negative T wave. Moreover, d-GAL-treated rats reported a significant increase in PR and QRS intervals by 18% and 176%, respectively as compared to the control group. In addition, heart rate significantly increased by 26%, as compared to the normal control group (Table 1).

**Figure 3:**
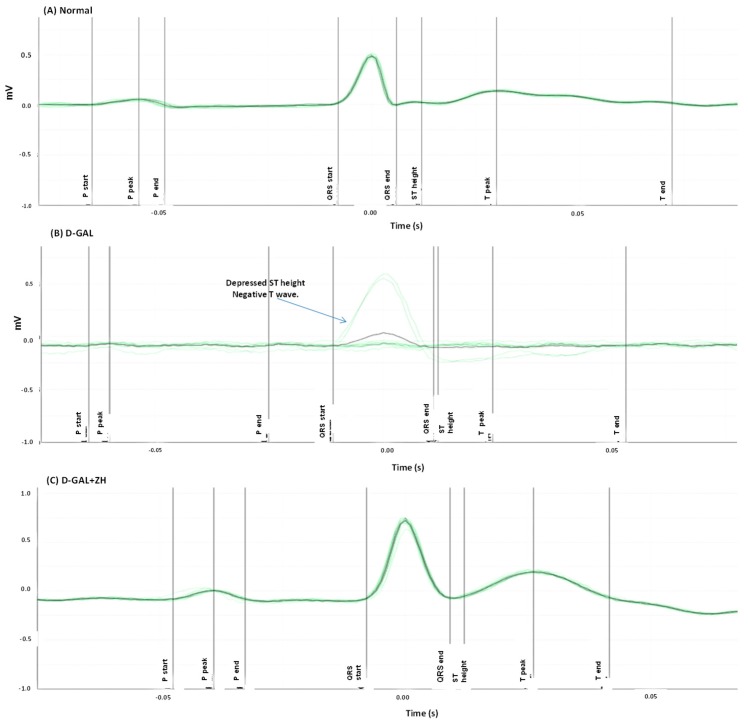
Effect of ZH on electrocardiographic (ECG) patterns in cardiac dysfunction induced in rats. ECG of normal rats showed a normal pattern (**A**). d-GAL treated rats showed an irregular rhythm of heartbeats and depressed ST height and negative T wave (**B**). d-GAL injected rats were orally treated with ZH for two weeks after d-GAL injection (**C**). The green lines represent the cumulative ECG pattern of a rat while the black line represents the average ECG pattern of a rat during 5 s.

Oral treatment of d-GAL-treated rats with ZH (250 µg/kg) showed an ameliorated ECG pattern in ST height and T wave (Figure 3). Treatment of d-GAL-treated rats with ZH (250 µg/kg) showed a decrease in PR interval and ST height by about 22% and 88%, respectively. Treatment with ZH (250 µg/kg) showed restoration in the QRS interval by an average of 38%. Heart rate was also attenuated by treatment with ZH (250 µg/kg) by about 30% (Table 1).

##### Effect of ZH on Biomarkers Measured in the Serum

d-GAL induced cardiac dysfunction was accompanied by an increase in serum levels of relevant markers; HS, CK-MK and LDH by about 2.7, 9, and 2.5 fold, respectively as compared to the normal control group. Oral administration of ZH (250 µg/kg) for 2 weeks post to cardiac dysfunction-induction showed a decreased serum HS level by about 53%. Similarly, serum CK-MK level and LDH level were reduced by an about 63%and 43%, respectively (Table 2).

##### Effect of ZH on Biomarkers Measured in the Cardiac Tissue

Cardiac dysfunction was associated with a marked decrease in the cardiac GLUT-4 by about 61% as compared to normal control. Treatment of cardiac dysfunction with ZH (250 µg/kg) up leveled by about 70% as compared to d-GAL treated group (Table 2).

**Figure 4:**
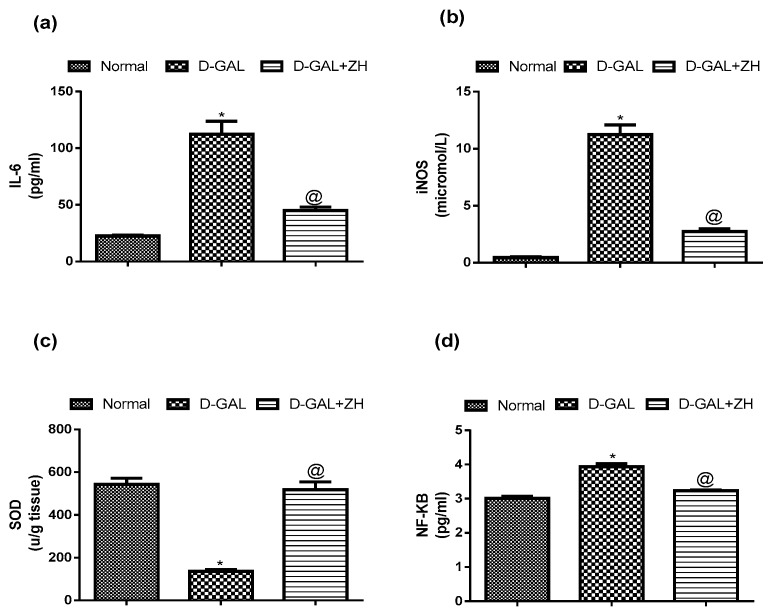
Effect of ZH on cardiac levels of interleukin-6 (il-6) (**a**), inducible nitric oxide synthase (iNOS) (**b**), superoxide dismutase (SOD) (**c**) and NF-κB (**d**) in cardiac dysfunction induced in rats. Data are presented as mean ± SEM. Statistical analysis was performed by one-way analysis of variance (ANOVA) followed by Tukey-Kramer test for multiple comparisons (*n* = 6–8). * significantly different from the normal group at *p* ≤ 0.05. ^a^ significantly different from d-GAL-treated group at *p* ≤ 0.05.

A marked elevation in the cardiac levels of IL-6 and iNOS reaching about 5 and 22 folds of the normal value, respectively was noticed in d-GAL injected rats. Oral administration of ZH (250 µg/kg) decreased the cardiac level of IL-6 and iNOS by 61% and 73%, respectively; compared to d-GAL treated group (Figure 4).

Induction of cardiac dysfunction by d-GAL in this study showed a significant increase in cardiac levels of SOD and NF-κB by about 74% and 30%, respectively, as compared to the control group. Oral treatment of d-GAL injected rats with ZH (250 µg/kg) restored the cardiac SOD content to nearly the normal value and ameliorated the cardiac NF-κB level by about 18%, as compared to d-GAL treated group (Figure 4).

##### Effect of ZH on RAR-α Expression

**Figure 5:**
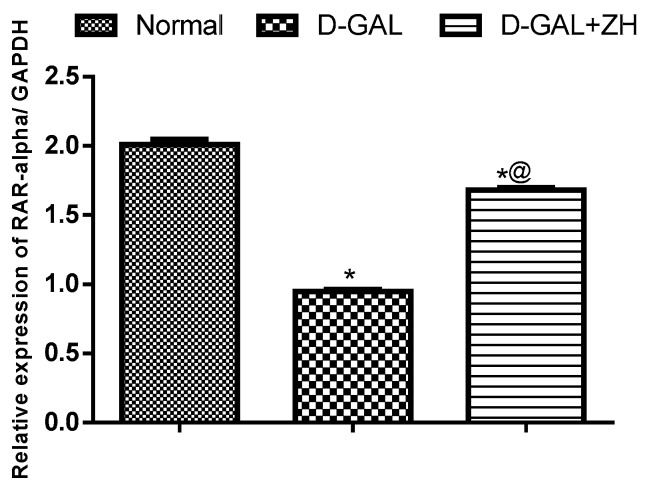
Effect of ZH on RAR-α cardiac expression in d-GAL-induced cardiac dysfunction in rats. Data are presented as mean ± SEM. Statistical analysis was performed by one-way analysis of variance (ANOVA) followed by a Tukey-Kramer test for multiple comparisons (*n* = 6–8). * significantly different from normal group at *p* ≤ 0.05. ^a^ significantly different from d-GAL-treated group at *p* ≤ 0.05.

d-Gal induced cardiac dysfunction is associated with a significant down-regulation in RAR-α gene expression the cardiac tissue reaching about 47% as compared to the normal control group. However, oral administration of ZH is associated with the up-regulation in the cardiac gene expression of RAR-α by 77%, as compared to d-GAL treated group (Figure 5).

##### Effect of ZH on the Histopathological Changes

**Figure 6:**
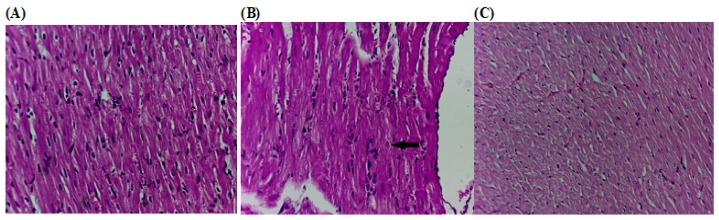
Effect of ZH on the histopathological alterations of the cardiac tissue of d-GAL-induced cardiac dysfunction in rats. (**A**) Control rat showing normal histological architecture (H and E × 300). (**B**) d-GAL treated rats (200 mg/kg; i.p.) showed an irregular rhythm of heartbeats. (**C**) Treatment of d-GAL-treated rats d-GAL injected rats were orally treated with ZH (250 µg/kg) for four weeks after d-GAL injection.

Histopathological examination of sections of cardiac tissues isolated from d-GAL-treated rats showed an abnormal myocardial architecture, decreased cellular volume with an exhibition of spaces between the cells. However, tissues isolated from rats treated ZH showed few spaces in between the cardiac myocytes but with apparently no pathological changes (Figure 6).

#### 2.3.3. Biochemical and Behavioral Safety Assessment

Liver and kidney functions were monitored at the end of the experiment to assess the safety of the treatment. As expected, induction of aging showed an elevation in levels of serum liver and kidney functions namely; ALT, AST, urea and creatinine by about 2.5, 1.5, 1.2 and 1.8 folds, respectively as compared to the normal control group. Treatment of d-GAL injected rats with ZH (250 µg/kg) has succeeded to attenuate the serum levels of liver and kidney functions to nearly the normal values (Table 3).

ZH treated group showed normal activity comparable to that of the normal untreated group, unlike d-GAL treated group which exhibited retarded activity. No convulsions or abnormal secretions were observed in any of the experimental groups. Water and food intake were more or less similar in all groups as well.

## 3. Discussion

*Dunaliella salina* microalgae is considered a good candidate for promoting cardiovascular activity according to a previous work performed in our laboratory [8,9]. The carotenoid rich fraction of *D. salina* showed high efficacy in modulating d-GAL induced cardiac dysfunction which promoted for further investigation of its phytoconstituents. Chromatographic analysis was performed resulting in the isolation of an orange amorphous powder which was identified according to spectral analysis as ZH (Figure 1) which was confirmed through comparison with previously reported spectral data [10]. Zeaxanthin, one of the most common carotenoids found in nature, is an anti-oxidant that accumulates in the retina of the human eye and protect the retinal structure from light-induced damage. It has been observed that consumption of diets with higher levels of zeaxanthin are accompanied by a low incidence of eye diseases such as age-related macular diseases, cataract, and diabetic retinopathy [11]. Currently, the possible role of ZH on cardiac remodeling process in elderly rats was investigated.

Age-associated cardiac dysfunction was associated with dramatic changes in the ECG pattern and a prominent increase in the serum cardiac biomarkers levels as well as an elevation in cardiac oxidative stress and inflammatory mediators. Moreover, histopathological cardiac examination revealed prominent changes in cardiac tissue architecture. ECG pattern showed an irregular rhythm of heartbeats, depressed ST height, negative T waves as well as elevated PR and QRS intervals in respect to normal rats which is an indication of myocardial disease [12]. In addition, heart rate significantly was elevated together with an increase in serum levels of cardiac function-relevant markers *viz.* HS, CK-MK and LDH and decrease in the cardiac GLUT-4 content as compared to the control group.

Previous studies have shown that age-related changes in impulse propagation may be related to abnormalities in the pattern of ventricular activation [13]. Recent data has suggested that alterations in heart rhythm intervals during aging may be associated with fibrosis and hypertrophy [14,15].

In accordance with other studies, induction of age-associated cardiac dysfunction by d-GAL showed a prominent elevation in cardiac levels of IL-6, iNOS, SOD and NF-κB. Aging is extensively associated with an imbalance between reactive oxygen species (ROS) production on one hand and antioxidant activities and NO bioactivity on the other [16]. Furthermore, inflammatory cytokines such as IL-6 is involved in proinflammatory signaling in the aging cardiovascular system. IL-6 is increased in aged myocardium which promotes myocardial damage and matrix remodeling, including collagen deposition [17,18]. Previously, it has been shown that the activity of myocardial NF-kB is increased in elder rats, which is reported to be associated with impairment of cardiomyocyte relaxation [18,19].

Zeaxanthin heneicosylate isolated from *D. salina* exhibited a satisfactory safety profile in the acute toxicity test where the experimental animals survived a single oral dose of up to 1 g/kg. Furthermore, ZH showed a significant improvement in ECG pattern, ST height, T wave and PR interval. It also restored the heart rate and QRS interval to normal range. Serum levels of HS, CK-MK and LDH showed a significant decline as well as cardiac GLUT-4 content. Additionally, ZH succeeded to decrease the cardiac level of IL-6 and iNOS and restore the cardiac SOD content to nearly the normal value and ameliorate the cardiac NF-κB level as compared to d-GAL treated group. It has been reported that direct inhibition of NF-κB effectively inhibits cardiac hypertrophy and cardiac dysfunction associated with aging [20]. Furthermore, the histopathological examination emphasized the obtained results where the cardiac tissue isolated from ZH treated rats showed minimal pathological alterations with respect to that isolated from normal ones. Moreover, ZH had the ability to restore the elevated levels of ALT, AST, urea and creatinine as compared to the d-GAL injected rats. These results indicate that zeaxanthin may have a hepatoprotective role in aged rats in accordance with Xiao et al. (2014) [21], a study that has been performed on an alcoholic fatty liver disease model and indicates as well that it protects the kidneys from the detrimental consequences of aging. There were no behavioral alterations in the ZH treatment group which further assures the safety of ZH. A number of studies investigated the safety of zeaxanthin and concluded that it shows a considerable amount of safety data based on regulatory studies. Subchronic studies with mice and rats receiving beadlet formulations of high purity synthetic zeaxanthin in the diet at dosages up to 1000 mg/kg body weight/day, and in dogs at over 400 mg/kg body weight/day, produced no adverse effects or histopathological changes. Zeaxanthin did not cause any signs of fetal toxicity or teratogenicity in rats or rabbits at dosages up to 1000 or 400 mg/kg bw/day, respectively. A 52-week chronic oral study in Cynomolgus monkeys at doses of 0.2 and 20 mg/kg bw/day, mainly designed to assess accumulation and effects in primate eyes, showed no adverse effects. In a rat two-generation study, the no-observed-adverse-effect-level (NOAEL) was 150 mg/kg bw/day. In 2012, this dosage was used by EFSA (NDA Panel), in association with a 200-fold safety factor, to propose an Acceptable Daily Intake equivalent to 53 mg/day for a 70 kg adult. The requested use level of 2 mg/day was ratified by the EU Commission [22].

**Figure 7:**
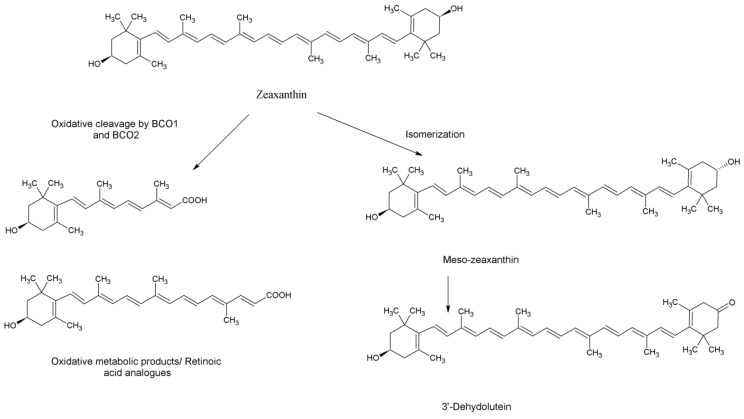
Metabolic products of zeaxanthin.

As far as we can find, there are three prospective describing the metabolic pathway of zeaxanthin in the mammalian body. The first postulation assumes that upon intestinal absorption zeaxanthin is incorporated in chylomicrons which are transferred to the liver, where they can be either stored or re-secreted into the circulation in association with lipoproteins [23]. The second approach suggests a comparative pathway to that of beta-carotene and the potential oxidative cleavage of zeaxanthin by β-carotene-15,15’-oxygenase (BCO1) or β-carotene-9’,10’-oxygenase (BCO2) into the apo-oxidation analogues of retinol and retinoic acid [2]. Finally, the third assumption involves an isomerization or oxidation at the position 3’which was based on the detection of meso-zeaxanthin and 3′-dehydro-lutein, respectively in human serum after supplementation with either lutein or zeaxanthin [24] (Figure 7).

In the course of investigating the effect on retinoic acid receptors, the oxidative metabolic analogues of retinoic acid; 15-apo and 9’-apozeaxanthenoic acids were studied for their affinity for RAR-α and RXR. Molecular docking of the 15-apo-carboxylic analogue of zeaxanthin showed high affinities towards RAR and RXR. The assumption that the pharmacological effect of ZH is mediated through the interaction with retinoid receptors was further confirmed through the evaluation of RAR-α gene expression which revealed a marked increase in the group treated with ZH as compared to the non-treated senile rats.

It has previously been shown that RA supplementation attenuates cardiac remodeling after experimental MI in rats [1] where RAR which are abundantly expressed in cardiomyocytes, were activated through heterodimerization with RXRs [25]. Studies showed that activation of RAR/RXR signaling prevented oxidative stress and apoptosis, in both neonatal and adult cardiomyocytes [26]. Recently, it has been reported that activation of RAR signaling prevented diastolic dysfunction, through inhibition of intracellular ROS generation and NF-κB signaling-mediated inflammatory responses [27].

These results proposed that RAR-mediated signaling has a major role in modulating cardiac oxidative stress due to pathological stimuli, which serves as an important mechanism in the development of diastolic dysfunction and heart failure. Experimental studies suggest that RA and similar ligands suppress both morphological alterations and changes in gene expression associated with hypertrophy which explains the effect of ZH [28]. Most studies that relate the role of RAR/RXR in the regulation of adult heart function have used RAR selective ligands or antagonists, due to heart malformation and poor survivability of genetic models of RAR deletion [29].

The findings of the current study are in line with the study by Iribarren et al., which revealed that the uptake of zeaxanthin was inversely related to the incidence of atherosclerosis [30]. Voutilainen et al. also pointed out that there is a positive correlation between the higher intake of fruits and vegetables rich in carotenoids and the prevention of morbidity and mortality with relation to CVD [31]. On the contrary, Bonds et al. reported that supplementation of zeaxanthin in addition to the daily intake of minerals and vitamins did not reduce the risk of CVD in elderly participants [32]. This may be attributed to the inclusion of subjects suffering from comorbid diseases; like diabetes and stroke, and other factors that can alter the outcomes *viz.* smoking. In addition, the study didn’t clarify the source of the used zeaxanthin; natural or synthetic which has an impact on the bioactivity. Despite the overall result of the study, it pointed out that there were fewer coronary revascularizations in the group receiving lutein and zeaxanthin (2.12%) versus the group receiving placebo (3.27%). Therefore, clinical investigation for the effect of a proper dose of natural zeaxanthin on cardiovascular disorders with the exclusion of any interfering factors for a sufficient period of time is recommended.

Finally, it can be concluded that ZH can be used as a natural therapeutic agent for ameliorating cardiac dysfunction exerting its action through the activation of retinoids receptors in cardiac tissue.

## 4. Materials and Methods

### 4.1. Cultivation of Dunaliella salina

*Dunaliella salina* was isolated from salt deposition basins of The Egyptian Salts and Minerals Company, EMISAL and grown on BG11 media [33] containing NaCl with a concentration of 100 g/L. The algal biomass was harvested and inoculated in plastic bottles with a capacity of 17 L containing 15 L of microalgae culture with continuous aeration. After growing for 10 days the culture was transferred to a fully automated and computer controlled photobioreactor with the capacity of 4000 L. Carbon dioxide was injected into the culture as a carbon source. The culture was left to grow until the biomass reached 2–2.5 g/L. Algal biomass was harvested by centrifugation at 2000 rpm and then sun-dried at 40–45 °C.

### 4.2. Preparation of Algal Extract

The dried biomass of *Dunaliella salina* was ground thoroughly for cell wall disruption. The biomass was extracted using hexane, ethyl acetate (80:20) till exhaustion. The extract was dried under reduced pressure in a rotary evaporator apparatus at a temperature not exceeding 40 °C until complete dryness.

### 4.3. Isolation Purification and Identification ZH

The algal extract was applied on top of a glass column dry-packed with silica gel and eluted with hexane with increasing proportions of ethyl acetate. Sub-columns for similar fractions were held for further separation of ZH. It was analyzed using NMR 1D and 2D (Bruker-400 MHz for ^1^H and 100 MHz for ^13^CNMR Spectrometer, (Bruker Corporation, MA, USA), mass spectrometer (EI-Finnigan MAT–TSQ 70 ev, (Thermo Finnigan, MA, USA) and ESI Bruker Daltonic Esqire-LC Amazon SL Ion Trap Mass Spectrometer (Bruker Corporation, Bremen, Germany), UV-visible spectrophotometer (Shimadzu UV 240, PIN 204-5800, Kyoto, Japan).

### 4.4. Docking Study

Docking calculations were carried out using Docking Server [34]. The MMFF94 force field was used for energy minimization of ligand molecules; 15-apo-zeaxanthen-15-oic acid and 9’-apo-zeaxanthenoic acid using Docking Server. Gasteiger partial charges were added to the ligand atoms. Non-polar hydrogen atoms were merged, and rotatable bonds were defined. Docking calculations were carried out on retinoic acid receptor (RAR) and retinoid X receptor (RXR) protein models. Essential hydrogen atoms, Kollman united atom type charges, and solvation parameters were added with the aid of AutoDock tools [35]. Affinity (grid) maps of 20 × 20 × 20 Å grid points and 0.375 Å spacing were generated using the Autogrid program. AutoDock parameter set- and distance-dependent dielectric functions were used in the calculation of the van der Waals and the electrostatic terms, respectively. Docking simulations were performed using the Lamarckian genetic algorithm (LGA) and the Solis and Wets local search method [36]. Initial position, orientation, and torsions of the ligand molecules were set randomly. Each docking experiment was derived from 10 different runs that were set to terminate after a maximum of 250,000 energy evaluations. The population size was set to 150. During the search, a translational step of 0.2 Å, and quaternion and torsion steps of five were applied.

### 4.5. Pharmacological Study

#### 4.5.1. Animals

Male Wistar albino rats weighing 130–150 g were obtained from the Animal House Colony of the National Research Centre, housed in plastic cages containing wood shavings, and kept under conventional conditions. The rats were provided with a basal diet and water ad libitum and allowed to acclimatize to the laboratory environment for 7 days before starting the experiment. The experiment was conducted in accordance with ethical procedures approved by the National Research Centre (Dokki, Giza, Egypt)—Medical Research Ethics Committee for the use of animal subjects.

#### 4.5.2. Chemicals

d-Galactose (d-GAL) was purchased from Sigma-Aldrich (St. Louis, MI, USA). All other chemicals used were purchased from standard commercial suppliers and were of analytical grade quality.

#### 4.5.3. Acute Toxicity Study 

The highest dose method was adopted for the acute toxicity test [37]. Mice were randomly assigned to one of two groups: A control group of six mice and a medicated group of 12 mice (six per cage, segregated by gender). The medicated group received a dose of 1 g/kg of the drug by oral gavage once. The control group was administered distilled water. Mice will have ad libitum access to food and water. The mice were observed for general behavior changes, toxicity and mortality continuously for 4 h after dosing, intermittently during a 24 h period and then kept for a further 14 days.

#### 4.5.4. Age-associated Cardiac Dysfunction Study

##### Experimental Design

Induction of age associated cardiac dysfunction in rats was carried out by intraperitoneal injection with d-GAL (200 mg/kg/day) for 8 weeks. Rats were divided into three groups, six rats each. Group I served as a negative control group, group II received d-GAL for 8 weeks and served as a positive control. Groups III received zeaxanthin ester from *D. salina*, (ZH; 250 µg/kg; p.o.), orally for four weeks after 8 weeks d-GAL injection. The dose was adjusted according to the dose used for β-carotene in rats (El-Missiry and Shalaby, 2000).

##### Electrocardiographic (ECG) Measurements

Twenty-four hours after the last dose of the drug administration, the rats were anaesthetized with thiopental (5 mg/kg, i.p.) and kept warm with a heating lamp to prevent the incidence of hypothermia. Subcutaneous peripheral limb electrodes were inserted for ECG recording (HPM 7100, Fukuda Denshi, Tokyo, Japan) and heart rate, PR interval, RR interval, QT interval, ST height as well as QRS duration were monitored.

##### Serum Collection and Tissue Preparation

At the end of the experiment, blood samples were collected from the retro-orbital sinus of the rats using non-heparinized capillary tubes for serum separation. Sera were then separated and stored at −20 °C as aliquots for further biochemical analysis. The rats were then sacrificed by cervical dislocation and hearts and kidneys were rapidly excised, washed with ice-cold saline, dried and weighed. Heart and renal tissues were then homogenized in 5–10 mL cold buffer per gram of tissue, using a homogenizer (HeidolphDiax 900, Germany) to prepare 10% homogenate. The resulting supernatant was transferred to Eppendorff tubes and stored at −80 °C until used for various biochemical assays.

##### Serum Biochemical Analysis

Sera stored at −20 °C were used for the estimation of serum cardiac and renal injury markers such as CK-MB, LDH. Serum CK-MB activity was assessed with the immune-inhibition method developed by [38]. LDH activity was determined enzymatically [39]. Serum levels of homocysteine (HS) were determined using enzyme-linked immunosorbent assay kit according to the manufacture procedures.

##### Cardiac Biochemical Analysis

Heart tissues were dissected out immediately, washed with ice-cold saline, homogenated and the homogenate was used for the determination of cardiac levels of superoxide dismutase (SOD), interleukin-(IL-6), inducible nitric oxide synthetase (iNOS) and GLUT-4 which were evaluated using enzyme-linked immunosorbent assay kit according to the manufacture procedures.

##### Assessment of Retinoic Acid Receptor-α Expression

Isolation of total RNA: Total RNA was isolated from cardiac tissues by standard TRIzol^®^ Reagent extraction method (Thermo-Fisher Scientific, Bremen, Germany). The extracted RNA was dissolved in diethylpyrocarbonate (DEPC)-treated water by passing solution several times through a pipette tip. Aliquots were used immediately for reverse transcription (RT), otherwise stored at −80 °C

Reverse transcription (RT) reaction: The complete Poly(A)^+^ RNA isolated from cardiac tissues was reverse transcribed into cDNA in a total volume of 20 µL using RevertAid™ First Strand cDNA Synthesis Kit (MBI Fermentas, Dreieich, Germany). The obtained cDNA was used for DNA amplification through real-time polymerase chain reaction (RT-PCR) [40].

Real time-Polymerase Chain Reaction (RT-PCR): StepOne™ real-time PCR System from Applied Biosystems (Thermo Fisher Scientific, Waltham, MA, USA) was used to determine rat’s heart cDNA copy number. PCR reactions were set up using SYBR^®^ Premix Ex TaqTM (TaKaRa, Biotech. Co. Ltd., Shiga, Japan). The sequences of specific primers of the RAR-α gene used are listed in Table 4. At the end of each qPCR, a melting curve analysis was performed at 95.0 °C to check the quality of the used primers. The relative quantification of the target to the reference was determined by using the 2^−ΔΔCT^ method [41].

##### Preparation of Sections for Histopathological Examination

After sacrifice, cardiac tissues were dissected out and samples were excised from the experimental animals of each group and fixed in 10% formalin. Tissues were then embedded in paraffin subsequently; 5 µM sections were cut on a microtome and examined microscopically for the evaluation of histopathological changes.

#### 4.5.5. Biochemical and Behavioral Safety Study

Serum levels of aspartate aminotransferase (AST) and alanine aminotransferase (ALT) were determined according to the methods of Reitman and Frankel [42]. The absorbance was measured at 510 nm. Serum Cr was determined according to the method of Larsen et al. (1972) [43] and the absorbance was measured at 459 nm. Serum urine was determined according to the method of Fawcett and Scott (1960) [44] and the absorbance was measured at 550 nm. General behavior including activity, grooming and convulsions as indicators of the central nervous system as well as daily intake of food and water were observed.

#### 4.5.6. Statistical Analysis

Data are presented as mean ± S.E. Statistical analysis of the data was carried out using one-way analysis of variance (ANOVA) followed by Tukey-Kramer’s multiple comparisons test to judge the difference between the various groups. Statistical significance was acceptable to a level of *p* < 0.05. Data analysis was accomplished using the software program GraphPad Prism (version 5, GraphPad Software, CA, USA).

## Figures and Tables

**Table 1:** Effect of zeaxanthin heneicosylate (ZH) on the heart rate, PR, RR, QT and QRS intervals and ST height in d-galactose (GAL) induced cardiac dysfunction in rats.

Group	Heart Rate Beat/Min	PR Interval (s)	QRS Interval (s)	ST Height (mV)
Normal	336.0 ± 10.26	0.0487 ± 0.0016	0.0128 ± 0.0002	0.0241 ± 0.002
d-GAL	422.4 ± 16.56 *	0.0576 ± 0.0028 *	0.0353 ± 0.0012 *	−0.0838 ± 0.010 *
d-GAL+ ZH	293.8 ± 14.47 ^a^	0.0448 ± 0.0020 *^,a^	0.0216 ± 0.0021 *^,a^	−0.0097 ± 0.014 *^,a^

Data are presented as mean ± SEM. Statistical analysis was carried out by one-way analysis of variance (ANOVA) followed by Tukey-Kramer test for multiple comparisons (*n* = 6–8). * significantly different from normal control group at *p* ≤ 0.05. ^a^ significantly different from d-GAL-control at *p* ≤ 0.05.

**Table 2:** Effect of ZH on the levels of homocysteine, creatine kinase-MB (CK-MB), lactate dehydrogenase (LDH) and glucose transporter type 4 (GLUT-4) in induced cardiac dysfunction in rats.

Groups	Serum Homocysteine (U/L)	Serum CK-MB (U/L)	Serum LDH (U/L)	Cardiac GLUT-4
Normal	3.95 ± 0.34	88.18 ± 4.72	184.52 ± 143.9	10.65 ± 0.37
d-GAL	10.85 ± 0.16 *	797.06 ± 53.90 *	465.42 ± 34.79 *	4.13 ± 0.35 *
d-GAL + ZH	5.10 ± 0.35 ^a^	291.40 ± 22.38 ^a^	267.41 ± 20.63 ^a^	7.02 ± 0.74 ^a^

Data are presented as mean ± SEM. Statistical analysis was carried out by one-way analysis of variance (ANOVA) followed by Tukey-Kramer test for multiple comparisons (*n* = 6-8). * significantly different from the normal control group at *p* ≤ 0.05. ^a^ significantly different from d-GAL-control at *p* ≤ 0.05.

**Table 3:** Effect of ZH on the serum liver and kidney functions in induced cardiac dysfunction in rats.

Groups	Serum ALT (U/mL)	Serum AST (U/mL)	Urea (mg/dL)	Creatinine (mg/dL)
Normal	48.33 ± 0.62	88.68 ± 4.08	41.74 ± 1.01	0.54 ± 0.08
d-GAL	121.37 ± 10.66 *	140.7 ± 6.09 *	54.25 ± 4.4 *	0.98 ± 0.02 *
d-GAL + ZH	56.21 ± 5.22 ^a^	91.48 ± 6.88 ^a^	43.67 ± 0.92 ^a^	0.59 ± 0.02 ^a^

Data are presented as mean ± SEM. Statistical analysis was performed by one-way analysis of variance (ANOVA) followed by a Tukey-Kramer test for multiple comparisons (*n* = 6–8). * significantly different from normal group at *p* ≤ 0.05. ^a^ significantly different from d-GAL-treated group at *p* ≤ 0.05.

**Table 4:** Primers sequence used for RT-qPCR.

Gene	Forward	Reference (GenBank)
*RAR-α*	F: aca act tcc cgc ttt tca cc	BC099830.1
	R: ctt tcg tac atc ttg ccc cg	
*GAPDH*	F:cgg cta cta gcg gtt tta cg	AY340484.1
	R:aag aag atg cgg ctg act gt	

RAR-α: Retinoic acid receptor-α; GAPDH: Glyceraldehyde 3-phosphate dehydrogenase.
